# Complete genome sequence of *Pigmentibacter ruber* isolated from a human patient in Japan

**DOI:** 10.1128/mra.00133-24

**Published:** 2024-08-20

**Authors:** Masahiro Hayashi, Ayumi Niwa, Jun Yonetamari, Yoshinori Muto, Sodai Yokoyama, Motohiro Nakamura, Yuta Yokobori, Mizuki Ogawa, Rina Ichioka, Ryosuke Kikuchi, Hiroyuki Okura, Shinji Ogura, Nobuyuki Tetsuka, Hisashi Baba, Kaori Tanaka

**Affiliations:** 1 Institute for Glyco-core Research iGCORE, Gifu University, Gifu City, Gifu, Japan; 2 Division of Anaerobe Research, Life Science Research Center, Gifu University, Gifu, Japan; 3 Gifu University Center for Conservation of Microbial Genetic Resource, Gifu, Japan; 4 Division of Clinical Laboratory, Gifu University Hospital, Gifu, Japan; 5 United Graduate School of Drug Discovery and Medical Information Sciences, Gifu University, Gifu City, Gifu, Japan; 6 Department of Cardiology, Gifu University Graduate School of Medicine, Gifu, Japan; 7 Department of Emergency and Disaster Medicine, Gifu University Graduate School of Medicine, Gifu, Japan; 8 Department of Infection Control, Gifu University Graduate School of Medicine, Gifu, Japan; 9 Center for Nutrition Support and Infection Control, Gifu University Hospital, Gifu, Japan; University of Maryland School of Medicine, Baltimore, Maryland, USA

**Keywords:** *Pigmentibacter ruber*, clinical isolate

## Abstract

*Pigmentibacter ruber* is a newly described bacterium belonging to the Silvanigrellaceae family that was isolated from human blood in 2021. We report the complete genome sequence of a clinical isolate of *P. ruber* (GTC16762) obtained from a human patient in Japan. Its genome contains a 3.6-Mb chromosome and three circular plasmids.

## ANNOUNCEMENT

*Pigmentibacter ruber* (Silvanigrellaceae family) is a Gram-negative, rod-shaped, aerobic, non-motile, pleomorphic bacterium with red pigmented colonies (1, 2). *P. ruber* GTC16762 was isolated from a 78-year-old male Japanese patient with necrotizing fasciitis and septicemia in 2021 (3). He was receiving corticosteroid treatment for erythroderma and presented to his previous physician with symptoms of fever, redness, and left lower extremity swelling. A pares of blood culture collected on admission tested positive after 3 days. The blood culture medium was centrifuged at 1,200 × *g* for 10 minutes, and the sediment was used for subculture with trypticase soy agar with 5% sheep blood (Nippon Becton Dickinson Co., Ltd.) and Chocolate II agar (Nippon Becton Dickinson Co., Ltd.) at 35°C under aerobic conditions and at 35°C under 5% CO_2_, respectively. Pink, smooth-shaped colonies were observed after 72 hours. 16S rRNA gene sequencing determined by Sanger sequencing prior to genome sequencing confirmed the presence of the Silvanigrellaceae family.

Bacteria were grown in trypticase soy agar with 5% sheep blood (Nippon Becton Dickinson Co., Ltd.) at 35°C under aerobic conditions for 72 hours. The colonies were scraped, suspended in TE buffer, and pelleted by centrifugation. Genomic DNA was extracted from the pellet using NucleoBond HMW DNA (TAKARA, Japan).

The entire genome of the isolate was sequenced using long-read sequencing by Oxford Nanopore Technologies (Tokyo, Japan) and short-read sequencing by DNBSEQ (MGITech Co., Ltd., Shenzhen, China) (4, 5). For long-read sequencing, a library was constructed using a ligation sequencing kit (SQK-LSK-109; Oxford Nanopore Technologies [ONT]) without shearing. Small DNA fragments were removed using a Short Read Eliminator (Pacific Bioscience of California, Inc, USA), and sequencing was performed using GridION X5 (ONT) on a FLO-MIN106 flow cell. Long-read sequence data were base-called with Guppy v.5.0.11. The raw reads were trimmed and quality-filtered using NanoFilt v.2.7.1 (https://github.com/wdecoster/nanofilt). For short-read sequencing, the MGIEasy FS PCR Free DNA library prep set (MGI Tech) was used for library construction. Subsequently, 150-bp paired-end sequencing was performed using DNBSEQ-G400 (MGI Tech). Raw sequencing reads were processed using fastp v.0.20.1 (6) with “-q 30 n 20 t 1 T 1” parameters. Short-read quality was checked using fastp v.0.20.1 (6), and the mean long-read quality was scored using NanoPlot 1.32.1 (7). High-quality short-read (over 89% of bases > Q30 averaged) and long-read (mean read quality of 14.4) sequences were assembled using Unicycler v.0.4.8 (8) with default settings. The assembly was rotated to start with the *dnaA* gene on the forward strand. The assembled contig graph was visualized using Bandage v.0.8.1 (9), and blobtools v.1.1 (10) confirmed the assembled genomic data integrity.

Quality assessment and genome statistics were computed using QUAST (v5.2.0) (7) and CheckM (v1.2.2), respectively (11). According to CheckM, the genome assembled in this study was 94.78% complete and 0.0% contaminated. The DDBJ Fast Annotation and Submission Tool (12) with default settings predicted 3,396 coding sequences, 15 ribosomal RNAs, and 43 transfer RNAs (Table 1). The closest relative of *P. ruber* GTC 16762 was *P. ruber* HNSRY-1 (GCF_009792895.1, average nucleotide identity value: 99.1) according to the Genome Taxonomy Database (GTDB, https://gtdb.ecogenomic.org).

**TABLE 1:** Information of the complete genome sequence of a *Pigmentibacter ruber* strain isolated from a Japanese patient

	Strain name
Parameter	*P. ruber* GTC16762
DNBSEQ sequencing^a^	
No. of reads	7,563,914
Size (kb)	1,134,587
Avg coverage (x)	293
DRA accession no.	DRR523861
ONT seqencing^a^	
No. of reads	100,547
Size (kb)	834,587
Avg read length (bp)	8,301
Avg coverage (x)	216
N50	13,113
DRA accession no.	DRR523860
Assembly	
Genome structure	One chromosome and three plasmids
DDBJ/GenBank accession no.	AP029159 (GTC16762)
(Chromosome/plasmid name)	AP029160 (pGTC16762-1)
	AP029161 (pGTC16762-2)
	AP029162 (pGTC16762-3)
	
Genome size (bp)	3614887 (GTC16762)
(Chromosome/ plasmid name)	140423 (pGTC16762-1)
	67953 (pGTC16762-2)
	44767 (pGTC16762-3)
GC content (%)	
(Chromosome/ plasmid name)	29.76 (GTC16762)
	29.89 (pGTC16762-1)
	27.75 (pGTC16762-2)
	31 (pGTC16762-3)
No. of coding sequences^b^	3,396
Number of rRNAs^b^	15
Number of tRNAs^b^	43
Number of CRISPRs^b^	2

^a^ DRA, DDBJ Sequence Read Archive.

^b^ DFAST, DDBJ Fast Annotation and Submission Tool. CRISPR: clustered regularly interspaced short palindromic repeats.

## Data Availability

The 16S rRNA sequence and genome sequences of the *P. ruber* (GTC16762) strain from human patient were deposited in DDBJ (https://www.ddbj.nig.ac.jp/index.html) with accession numbers LC795719 and AP029159-AP029162, respectively. Raw sequence data were deposited in the DDBJ/Sequence Read Archive under accession numbers DRR523860 and DRR523861 for GTC16762.
